# The Evaluation of Building Fire Emergency Response Capability Based on the CMM

**DOI:** 10.3390/ijerph16111962

**Published:** 2019-06-03

**Authors:** Guofeng Ma, Sheng Tan, Shanshan Shang

**Affiliations:** 1 School of Economics and Management, Tongji University, Shanghai 200092, China; guofengma@tongji.edu.cn; 2 School of International Business Management, Shanghai International Studies University, Shanghai 201620, China; sss336699@hotmail.com

**Keywords:** capability maturity model (CMM), fire emergency response capability evaluation, public safety, building information modeling (BIM), evaluation plug-in

## Abstract

The construction of smart cities is a theme of urban development, and building fires greatly threaten public safety and urban environmental governance, in which fire emergency management is one of the key factors. However, most studies on the evaluation of emergency response capacity ignore the process of improvement, as well as the intelligence and practicality of the results. The evaluation system of building fire emergency response capability maturity (FE-CMM) was innovatively proposed based on the capability maturity model (CMM), including the evaluation index, evaluation grade, evaluation method, and evaluation process. At the same time, a plug-in for evaluating fire emergency response capability was developed based on the building information modeling (BIM) platform. Finally, an empirical study was carried out in combination with the case of a district fire center. The research demonstrates that the evaluation system can effectively judge the maturity of fire emergency response capability, and the established plug-in can preliminarily realize the intelligent evaluation of building fire emergency response capability, which improves the practice and intelligence of the fire emergency response capability evaluation system when fully considering the process of improvement. It has guiding significance for ex ante control and refined management of building fires, thus providing support for urban public safety and environmental governance.

## 1. Introduction

Smart cities are gradually becoming the center of urban construction [1]. Public safety and environmental governance are important tasks in the construction of smart cities [2]. Building fires represent one of the most common emergencies in the city, which threatens urban public safety and environmental governance [3,4]. Citizens are more diverse and clustered, building structures and urban environments are becoming more complex, and the standardization of fire management is higher, all leading to the fact that urban fire prevention requires more attention, and fire rescue is becoming more complicated, thereby increasing the difficulty of urban fire emergency management [5,6]. The refined management and ex ante control of building fire is gradually becoming the focus of research with the development of science and technology to reduce the loss of life and property [7].

The existing research on fire emergency mainly focuses on three aspects: (1) researching new specific problems, (2) applying and optimizing new methods, and (3) focusing on the practicality and intelligence of the results, which is always reflected in system integration and platform development. Under the umbrella of new specific problems, Kwok et al. [8] highlighted the role of Vancouver Fire and Rescue Services in the coordinated hoarding response of the city. In order to better understand the movement during stairwell evacuations, Peacock et al. [9] provided stair movement data during fire drill evacuations of office and residential buildings to test the predictive capability of building egress models. In terms of new methods and method optimization, Zhang et al. [10] proposed a probability-based Monte Carlo life-risk analysis model to assess the probability of injury and death in fires. Cao et al. [11] presented an extended multi-grid model to study pedestrian movement and exit selection in fire. In terms of intelligence development, Chen et al. [12] proposed a building information model (BIM)-based visualization and warning system for fire rescue. Cheng et al. [13] built a BIM-based intelligent fire rescue system, which integrates personal positioning information, evacuation/rescue path optimization, and move boot device information. However, most researches ignored ex ante control of fire emergency.

Emergency response capability evaluation (ERCE) is essential to improve the ex ante control of fire emergency management. For example, Liao et al. [14] proposed a hesitant fuzzy linguistic preference utility TOPSIS (Technique for Order Preference by Similarity to an Ideal Solution) method to select the best fire rescue plan, whereby an emergency response evaluation could also help managers discover the best plan. However, the current ERCE studies ignore the fact that the improvement of emergency response capability is a gradual process, and the research results of ERCE lack practicality and intelligence to some extent, suggesting a need for improvement [15,16].

Maturity refers to the ability to characterize the development of things from a quantitative perspective. The capability maturity model (CMM) originated from software development management, which considers the processional nature of emergency management optimization [15]. Most maturity evaluation studies are limited to obtaining the maturity level, while the results of the CMM not only get the maturity level, but also more intuitively demonstrate the current and future tasks in the optimization path. The establishment of a fire maturity rating system based on CMM can solve the shortcomings of previous studies that ignored the improved process. As for the practicality and intelligence of the research results, building information modeling (BIM) technology has the advantages of information integration, full process control, and visualization, which provides technical support for the prevention and the control of potential hazards. In actual building fire rescue, more and more advantages of BIM technology are being used to guide the fire rescue, such as visual process management. Integrating a fire emergency response capability maturity (FE-CMM) system into a BIM platform can further realize information integration and facilitate fire rescue. Therefore, the establishment of a building fire rescue system based on CMM and its integration into the BIM platform can solve the problems that most previous research ignored, addressing the improved process and the weak practicality and intelligence of results [17].

Thus, in order to accurately obtain the maturity level of a fire emergency, this study proposes and establishes a new building fire emergency maturity evaluation system based on the CMM for the first time to solve the problems that most previous researches ignored. In order to improve the process and the practicality of the system, a BIM-based evaluation plug-in was developed to improve the intelligence of the system, so as to improve the practicality of the system. Finally, an empirical study was conducted according to data from a fire center in a certain area to demonstrate the usefulness of the system and plug-in. This paper provides researchers and fire management units (especially the fire department) with a continuous improvement method for fire emergency management, and contributes to urban public safety and environmental governance.

## 2. Literature Review

There is a lack of research on urban fire emergency response capability at present, and comprehensive research on the evaluation of emergency response capability in other fields illustrates that the methods have certain limitations, as shown in Table 1. One is that the commonly used qualitative methods are subjective and affect the accuracy of the results. For example, Ali et al. [18] conducted a risk assessment study on a large manufacturing company based on analytic hierarchy process (AHP) and the Fine–Kinney risk assessment method. Ju et al. [19] proposed a hybrid fuzzy method consisting of a fuzzy analytic hierarchy process and binary fuzzy language to evaluate the emergency response capability. Liu and Zhang [20] proposed a multi-objective location selection mode based on AHP theory to optimize emergency response time. Wu et al. [21] established an urban community fire ERCE system and comprehensive evaluation model based on a fuzzy analytic hierarchy process. However, to some extent, all the methods of these researchers are inherently subjective.

The second limitation is that some researches ignore the processional nature of emergency management improvement, which is usually reflected in the fact that the researches are usually limited to one-time measures to improve the emergency response level. For example, Zhou et al. [22] proposed a timed colored hybrid Petri net (TCHPN) method to evaluate different emergency response actions based on their efficiency in preventing or delaying the propagation of domino effects. Ke et al. [23] established an evaluation model of the emergency capability of earth-rock cofferdam construction. Jin et al. [24] established an evaluation index system for marine oil spill response capability based on an assessment of the advanced experience of domestic and foreign emergency capability, but also ignored the process of improvement of emergency management.

The third limitation is that the research results are often insufficiently combined with practice, and the intelligence of the results is not sufficient, which is not conducive to practical application and promotion. Ping et al. [25] presented a model to estimate the probability of successful escape, successful evacuation, and successful rescue on offshore platforms. Song et al. [26] built a measurable index system for the evaluation of campus emergency management capacity based on a principal component project method; however, they ignored the practicality of the improvement results. To some extent, all of the researches had complicated processes and insufficiently combined outcomes with practice and intelligence.

The CMM fully considers the improvement process, and some scholars tried using CMM to solve ERCE and achieved good results. Ji et al. [27] constructed a maturity model of emergency management capability evaluation for college emergencies based on CMM, and established a quantitative maturity level test method based on the variable weight method and fuzzy theory. Nie and Han [28] constructed an emergency logistics capability maturity model based on CMM, and analyzed the main influencing factors using a Bayesian network. At the same time, BIM technology is increasingly being used in emergency capability evaluation because it can improve the intelligence of results. Li et al. [29] introduced a BIM-centric environment-aware beacon deployment algorithm to locate first rescuers and trapped people in a building fire emergency scenario. Zang et al. [30] proposed a BIM-based immersive game environment method to study the interaction between people and buildings during evacuation. Cheng et al. [31] developed a simulation model based on BIM technology to evaluate different evacuation plans and improve the reliability of evacuation plans. 

In summary, some urgent problems still exist in the current researches on ERCE in various fields, such as insufficient practice and intelligence, and there is little research on urban building fire emergency response capability. The CMM and BIM technologies brought new ideas to ERCE research. Applying CMM and BIM technology to building fire emergency response capability evaluation is a direction which is worth further investigating.

## 3. Establishment of FE-CMM Evaluation Model

### 3.1. Maturity Level

Contemporary fire emergency management aims for reasonable and orderly processes, accurate and complete information, scientific and effective resource allocation, and efficient and rapid emergency response. These goals are also indispensable test conditions for fire emergency management. It is vital to establish normative systems and processes through all stages in emergency management to improve the efficiency. In addition, fire emergency management processes and systems require to be continuously optimized to adapt to increasingly complex fire emergencies [32]. Fire emergency processes are full of uncertainty and require prior control; thus, it is very important to obtain effective and sufficient information about emergency responses. Getting relevant information effectively and in a timely manner can greatly improve the rescue efficiency [33]. The success of emergency rescue depends on resources. Therefore, how to improve the probability of rescue success under resource constraints and how to balance the relationship with cost is one of the key points within the scientific development of fire emergencies [34]. The importance of fire emergency rescue time is self-evident, and a shortened emergency response time can greatly improve rescue efficiency [35]. Therefore, process management, information management, resource management, and time management are extracted as important distinguishing indicators. Moreover, the maturity is divided into initial level, growth level, standard level, quantifying level, and optimization level according to the degree of achievement of each indicator and the traditional software maturity model (Software Engineering Institute Capability Maturity Model, SEI-CMM), as shown in Table 2.

### 3.2. Key Process Identification and Refinement of Key Process Areas (KPA)

It is necessary to decompose the key processes, which can help subdivide the key process areas. According to the idea of OPM3 (Organizational Project Management Maturity Model) of the American Project Management Institute, the characteristics of fire emergency and its routine response process (i.e., the key processes in fire emergency) are divided into preparation process, identification process, execution process, control process, and post-emergency process. The content and objectives of each process are shown in Table 3. The five key processes are further decomposed to establish key process areas for each maturity level according to the characteristics of the five assessment dimensions of maturity classification (Table 2), and then the 21 evaluation indicators (key process areas) are condensed and combined with the current research results and fire emergency management actual characteristics. It should be noted that technology is the most direct indicator of characterization in the context of science and technology. Most studies focus on the technical characteristics when selecting indicators, and build specialized technology maturity [36,37]. At the same time, in consideration of the importance and extensiveness of BIM technology in fire emergency management, BIM technology is regarded as one of the key evaluation indicators of the quantifying level, which is independent of the technical application index [38]. The key process areas for each maturity level are shown in Table 4.

### 3.3. Defining the Target Set of Key Process Areas

The target set of each key process area was defined to help experts, fire management personnel, and article readers understand each key process area. It is necessary to define a criterion for each key process area with the above work completed. The criterion considers the content of the key process area and the degree of it to be achieved as the judgment standard of whether the organization effectively implemented the key process area. Therefore, the key evaluation indexes of each maturity level were designed through literature review and field research, as shown in Table 5, Table 6, Table 7 and Table 8 [39,40].

## 4. FE-CMM Evaluation Plug-In Design

**Figure 1:**
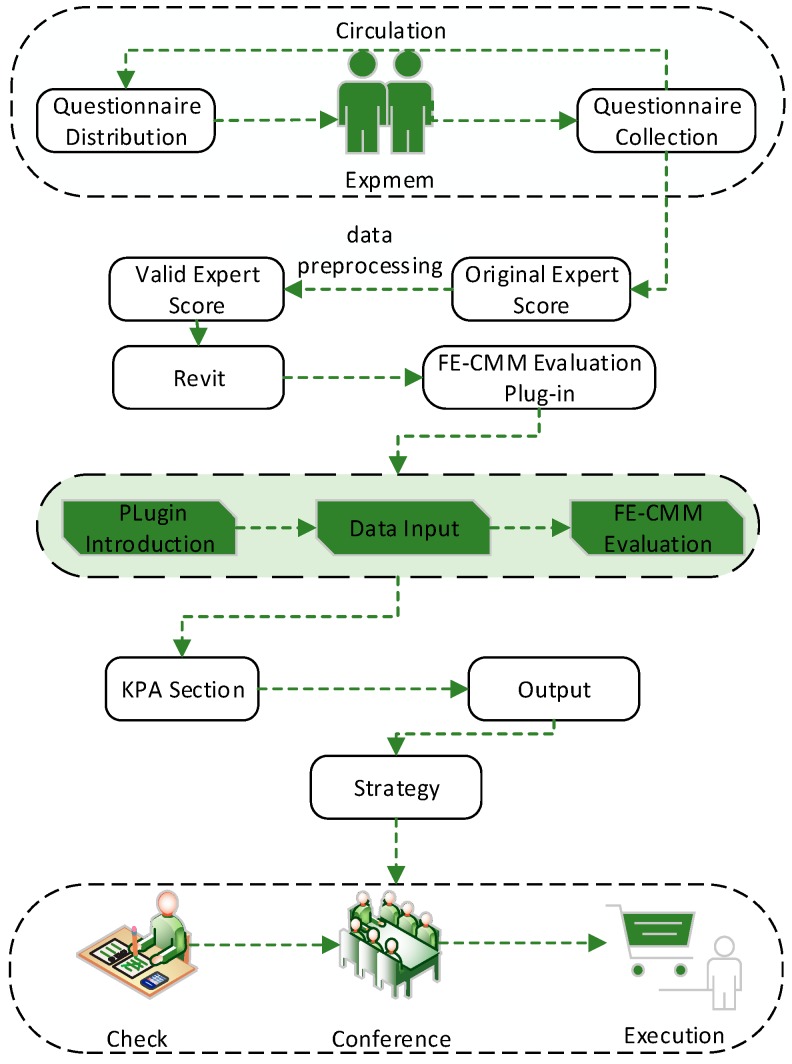
The fire emergency response capability maturity model (FE-CMM) evaluation data stream.

**Figure 2:**
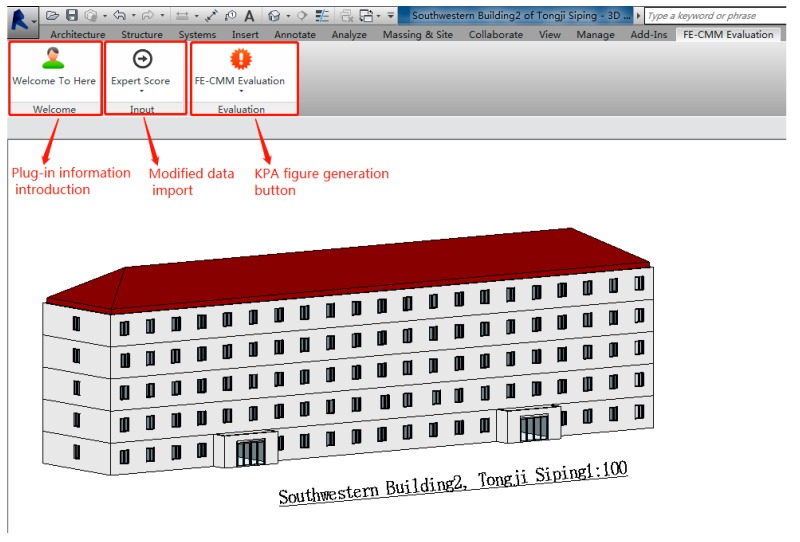
FE-CMM evaluation function.

To improve the intelligence and practicality of the FE-CMM system, a FE-CMM evaluation plug-in was developed with C# programming language based on the Revit 2016 software platform (Autodesk, San Rafael, CA, USA). Figure 1 depicts that, firstly, the researchers, such as the university or social fire emergency management investigation team or fire department, distribute the key process area implementation score questionnaire and research-related materials to college experts, the fire brigade, building security personnel, and other building fire-related management personnel, such as emergency mechanisms and emergency plans. The questionnaire is a disordered key process area that is to be scored, whereby experts grade each key process area based on provided material or practical experience. After collecting and counting survey questionnaires to obtain a key process area score, the user is required to import the key process area score into the plug-in using the “expert score” function. Next, the user can also modify it in the Revit software, and then click on the “FE-CMM evaluation” function to output the KPA profile. Then, the emergency manager obtains the fire emergency maturity level and the defects in the current emergency management according to the output KPA profile, which provides a targeted improvement direction for improving the fire emergency response maturity level. Figure 2 depicts three functions of the FE-CMM evaluation plug-in, including the introduction of the plug-in, data import, and the evaluation of the plug-in.

**Figure 3:**
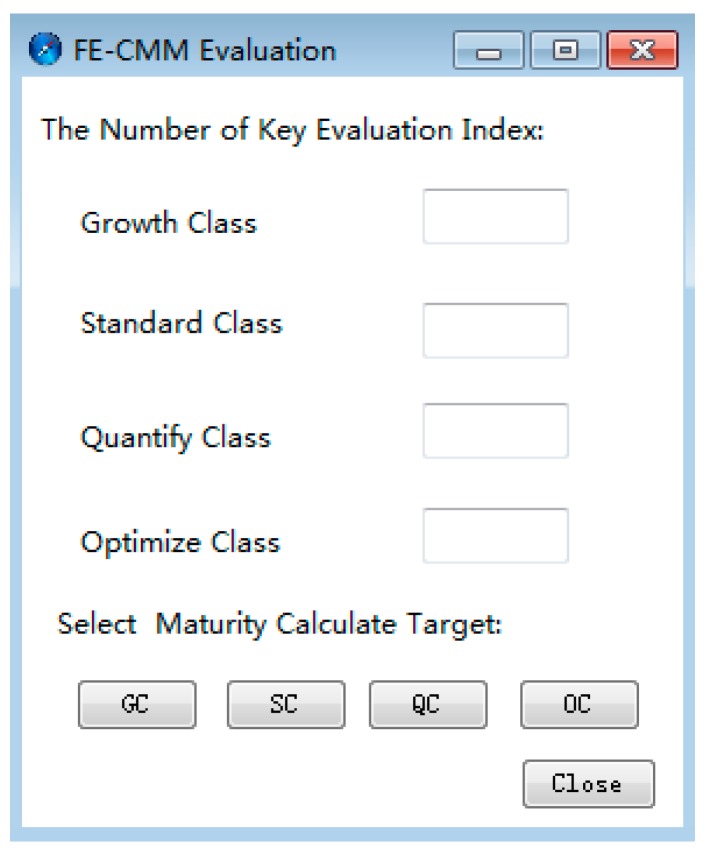
FE-CMM evaluation key process area (KPA) chart output interface.

As shown in Figure 3, upon clicking the FE-CMM Evaluation function of the plug-in, the KPA property setting interface will pop out. Then, upon entering the evaluation indicator number of each level, the plug-in will select the maximum number of evaluation indicators as the final row number of KPA charts suitable for all maturity levels. In addition, the user can choose a certain maturity level to output a KPA figure. However, the FE-CMM evaluation plug-in can only output one KPA map of maturity level at a time, instead of outputting KPA profiles of all maturity levels.

## 5. Empirical Research

In order to verify the feasibility and rationality of the fire FE-CMM evaluation system, a fire center was selected as the research object to evaluate the fire emergency response capability. The fire center is located in a relatively developed area where old-fashioned buildings account for a large proportion of the buildings. In addition, there are a great number of factories and enterprises; thus, the proportion of fires is higher than that in most similar jurisdictions. Therefore, the fire emergency response of the fire center is relatively more frequent, and the fire emergency experience is rich, which are the main reasons for the object selection.

To implement the research, a team of 14 experts, including 10 school-age research experts and four fire department managers, was organized at the beginning. The expert team was divided into two groups, due to the school enterprise research experts (group A) lacking experience in actual situations and the fire center management personnel (group B) lacking theoretical knowledge and objectivity. Group B serves as a reference group and plays a complementary role in fixing the result of group A, and this can effectively avoid the shortcomings of the two groups.

**Figure 4:**
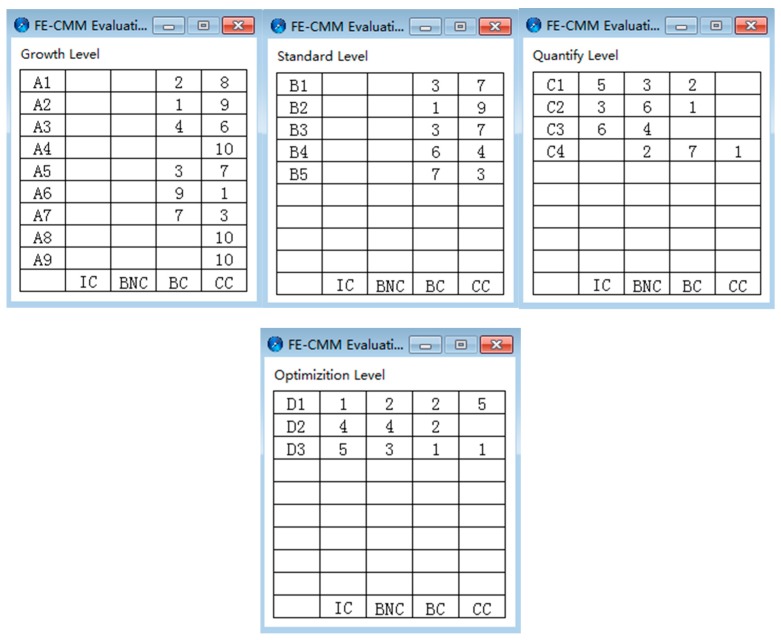
FE-CMM evaluation output (the result of group A).

**Figure 5:**
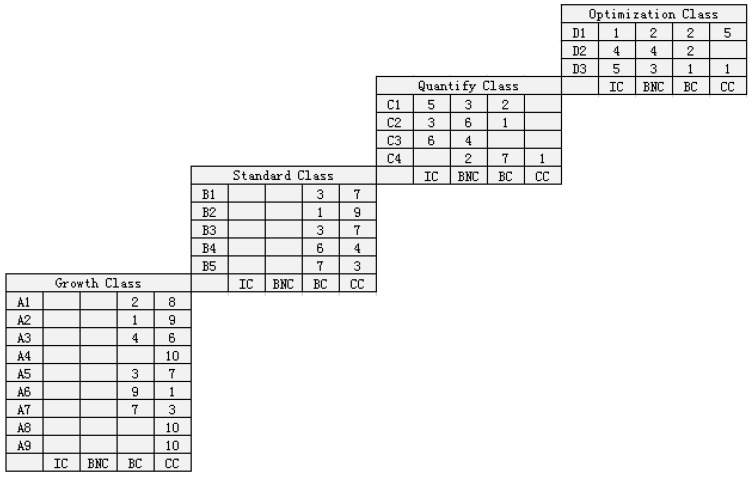
Fire emergency maturity KPA profile.

Group A judged the achievement degree of the 21 key process areas based on the provided materials, and there were four achievement degrees as follows: incompatible (IC), basically not compatible (BNC), basically compatible (BC), and completely compatible (CC). Group B assigned a specific score between 1 and 10 to each realization degree of the 21 key process area under the premise that they were unaware of the conversion indicator, because they were front-line managers. If they chose the achievement degree directly, they were easily influenced by the role and made non-objective judgments. Then, the investigator converted the score result into the achievement degree according to the indexes in Table 9. If the results of the key process areas from two groups were inconsistent and affected the final maturity level determination, there was a multi-party discussion to determine the achievement degree. Finally, as shown in Figure 4, the judgment chart of each maturity level could be obtained by importing the judgment result of group A into the plug-in, and Group B obtained a situation determination table, as shown in Table 10. As seen in Figure 5, the final KPA chart can be obtained through the above process, allowing the comparison of the results of the two groups.

**Figure 6:**
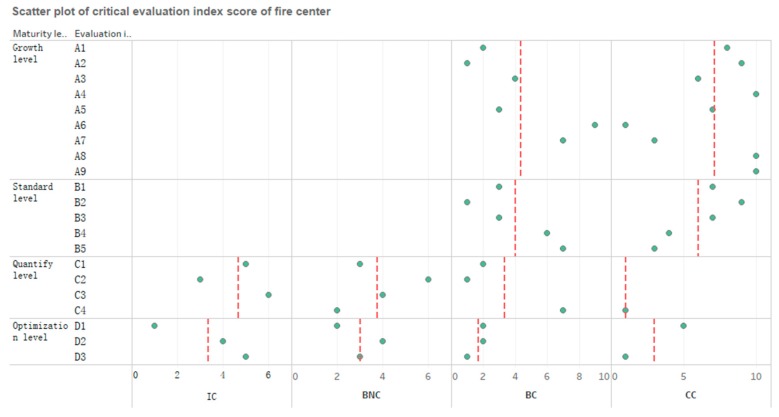
Scatter plot of critical evaluation index scores of the fire center.

Figure 6 is drawn in order to facilitate the analysis. The following six aspects of the findings were obtained through the aforementioned research of the FE-CMM system:

(1) There was no difference between the results obtained by group A and group B, which led to no difference in the fire emergency response maturity level. The KPA profile of group A had dual credibility of school enterprise research experts and fire-fighting center managers due to the IC and BNC not being shown in the results of A1–A9 and B1–B5.

(2) As can be seen in Figure 4 and Figure 5, the fire emergency response capability of the fire center was at a standard level. It can be seen more intuitively from Figure 6 that there were a few points located with IC and BNC in the abscissa, where the ordinates were growth level and standard level, while there were many points located at the area denoting the quantifying level and the optimization level for IC and BNC. This also indicates that the target maturity was at the standard level. 

(3) Although the maturity level of fire center reached the specification level, there were still problems in urgent need of a solution in B4 (fire drill) and B5 (summary promotion). In addition, A6 (technical use) and A7 (tracking monitoring) also require improvement in the growth class.

(4) There are still many problems to be handled if the maturity level is to reach the quantifying level. C1 (resource allocation), C2 (state of affairs control), and C3 (time management) require continuous improvement in the future, in addition to A6, A7, B4, and B5. In particular, the improvements of C1 and C3 require a lot of time and capital. There is no in-depth condition to grasp the emergency time and resources in a quantitative way, caused by an insufficient understanding of technology, quantitative rescue, and the inactive application of new technology.

(5) D1 performed well in the current optimization level, indicating that the fire department center made an effective adjustment in the rescue process after fire. However, in order to improve it to the optimization level, it is necessary to improve D2 (technical optimization) and D3 (preventive optimization), which are largely insufficient at present. 

(6) In addition, as can be seen in Figure 6, the dashed line shows the average score of the IC, BNC, BC, and CC at each maturity level. Under the premise of only focusing on the average number (the dashed line), the scores of IC and BNC increased, while the score of CC was reduced with the improvement of maturity level, which is reasonable. At the same time, the scores of IC and BNC at the growth level and standard level were zero according to Figure 6, which helps visualize the maturity level more, especially when there are a lot of evaluation indexes.

## 6. Conclusions

Building fire emergency management is a key factor of urban construction and public safety protection. The importance of implementing ex ante control is self-evident due to fires being often accompanied by a large number of losses. Therefore, this study focused on the evaluation of building fire emergency response capabilities, where the key contributions in this study are summarized as follows:

(1) Three main problems in the current research on emergency response capacity evaluation were discovered: the methods or the processes of some researches are subjective, the improvement process is neglected and unsustainable, and there is weak practicality of the research results.
(2) A building fire emergency response capability evaluation system was established based on CMM, which can accurately identify maturity levels, and guide the fire brigade, building security personnel, and other building fire-related management personnel to continuously improve and target building fire emergency response capabilities.
(3) A smart evaluation plug-in was developed based on the Revit software, which improves the practicality of the evaluation system and makes it easier for researchers or fire emergency management to use the evaluation system.

However, there are still some limitations to be improved in the research. Firstly, practical improvement is not given although the study designed the experimental process and experimental set-up in the empirical research to reduce the subjectivity of the evaluation. Secondly, the plug-in has a single function and requires further improvement.

## Figures and Tables

**Table 1:** The major methods for emergency response evaluation and their main features. CMM—capability maturity model; BIM—building information model.

The Major Methods for Emergency Response Evaluation	Main Features
The analytic hierarchy process	Subjective, ignores the improvement process, limited
Fuzzy comprehensive evaluation method	Subjective, ignores the improvement process
Fuzzy comprehensive analytic hierarchy process	Subjective, ignores the improvement process
G1 weighting method	Weak subjectivity, ignores the improvement process, weak practicality
Artificial neural network	Weak subjectivity, ignores the improvement process, weak practicality
CMM + BIM	Subjective, highlight the improvement process, high practicality
Catastrophe theory, case-based learning, cloud model	Weak subjectivity, ignores the improved process, weak practicality

**Table 2:** Fire emergency response (FE)-CMM classification.

Grade	General Characteristics	Process Management	Information Management	Resource Management	Time Management
Initial level	The fire emergency process is disordered, and the method is rough	Lack of emergency rescue process	Information clutter, lack of information acquisition, and application capabilities	Resource management is confusing; resource decision-making and deployment rely on personal feelings	Insufficient knowledge about the rescue time, and no control over it
Growth level	Establishes a basic fire emergency response process; however, the process is not very normative	The emergency process is fixed and more effective	Establish a more stable information management system; the ability to access and apply information	Establish a basic resource management system and make decisions and allocate resources according to basic principles	Ability to preliminarily judge temporal needs and priorities based on experience
Standard level	Standardization of fire emergency procedures	The emergency process is intact, and a written document is formed	Ability to effectively obtain the required information and use it to guide rescue	Resource management forms a fixed method and process, and resource allocation and decision-making follow	Can better control time and standardize operations
Quantifying level	The process can be controlled by a quantitative method	Metrics are established and the process can be quantitatively analyzed	Compare the information acquisition and application of methods; analyze existing problems	Use quantitative methods to assist resource allocation and decision-making	Control the optimal time and actual time according to the quantitative method
Optimization level	Ability to use science and technology to optimize processes	Quantitative analysis results and new techniques are applied for process optimization	Information dynamic tracking, storage, continuous optimization of information acquisition, and application	Optimize resource allocation continuously and dynamically to improve the scientific decision-making of resources	Ability to continuously optimize and spend the actual time spent

**Table 3:** Key processes of fire emergency rescue.

Process Name	Goals and Content
Preparation process	Conduct fire monitoring, fire prevention publicity, and fire equipment maintenance inspection
Identification process	Identify the occurrence of a fire and obtain specific information about the fire
Execution process	Fire rescue resources according to fire information for fire rescue and suppression
Control process	Monitor and rescue operations, analyze and make decisions about emergencies, and promote the achievement of goals
After the process	Implement post-disaster recovery, summarize fire occurrences and emergency rescues, and guide future rescues

**Table 4:** Key process areas of each maturity level.

Initial Level	Growth Level	Standard Level	Quantifying Level	Optimization Level
	A1. Inspection system	B1. Emergency plan	C1. Resource allocation	D1. Process optimization
	A2. Team building	B2. Organization and coordination	C2. Situation control	D2. Technical optimization
	A3. Information transfer	B3. Conscious promotion	C3. Time management	D3. Preventive optimization
	A4. Disaster location	B4. Fire drill	C4. BIM application	
	A5. Disaster information	B5. Summary and promotion		
	A6. Technical application			
	A7. Tracking monitoring			
	A8. Emergency command			
	A9. Cost management			

**Table 5:** Growth-level key process area objectives.

Evaluation Index	Description
A1	Distribute specialized personnel to regularly investigate areas where fire safety hazards may exist
A2	The rescue workers are selected according to the standard, the duties of the personnel are clear, and the actions are responsible
A3	Establish an effective way to accurately, quickly, and completely transfer information to the relevant personnel
A4	Can quickly obtain the location of the fire, and have a general understanding of the surrounding traffic and residents after the fire
A5	Can quickly obtain fire-related information, including fire type, fire grade, and so on, after a disaster occurs
A6	Ability to apply or develop new fire emergency rescue technologies
A7	Have an understanding of rescue situations and fire conditions during the rescue process
A8	An emergency command team can be established, and emergency command can be carried out according to the specified procedures after the disaster occurs
A9	There is an emergency rescue fund which can be put into emergency rescue

**Table 6:** Standard-level key process area objectives.

Evaluation Index	Description
B1	Have an emergency plan, and its preparation in strict accordance with the standard, which can be put into use according to the process
B2	In fire rescue, all involved departments or personnel act in an orderly and responsible manner
B3	Enhance the awareness of fire prevention and fire knowledge according to the procedures and standards
B4	Hold fire drills regularly
B5	Hold a summary meeting, with an analysis and discussion of experience and lessons, and promote these after the fire

**Table 7:** Quantifying-level key process area objectives.

Evaluation Index	Description
C1	Guide the allocation of fire resources according to experience or technical after the fire
C2	Ability to take effective control processes to keep the fire under control
C3	Ability to effectively determine the rescue time required
C4	Can use BIM technology to assist emergency rescue

**Table 8:** Optimization-level key process area objectives.

Evaluation Index	Description
D1	Adjust the fire emergency rescue process according to the summary
D2	Upgrade the technical of rescue
D3	Supplement and adjust the measurement

**Table 9:** Implementation status conversion indicators. IC—incompatible; BNC—basically not compatible; BC—basically compatible; CC—completely compatible.

Realization Situation	IC	BNC	BC	CC
Score interval (minutes)	0–3	4–6	6–8	9–10

**Table 10:** Fire emergency maturity judgment score.

Evaluation Index	Comprehensive Score	Implementation Level
A1	9.25	CC
A2	9.25	CC
A3	9	CC
A4	9.5	CC
A5	8.75	BC
A6	8.5	BC
A7	8.75	BC
A8	9.75	CC
A9	9.5	CC
B1	8.25	BC
B2	9	CC
B3	8	BC
B4	7	BC
B5	6.75	BC
C1	3.5	BNC
C2	5.75	BNC
C3	3.5	BNC
C4	7.25	BC
D1	7	BC
D2	3	IC
D3	3.25	BNC
